# CD80 Insights as Therapeutic Target in the Current and Future Treatment Options of Frequent-Relapse Minimal Change Disease

**DOI:** 10.1155/2021/6671552

**Published:** 2021-01-06

**Authors:** Yoong Mond Teh, Soo Kun Lim, Norhana Jusoh, Kahar Osman, Siti Aisyah Mualif

**Affiliations:** ^1^School of Biomedical Engineering and Health Sciences, Faculty of Engineering, Universiti Teknologi Malaysia (UTM), Johor Bahru, Malaysia; ^2^Renal Division, Department of Medicine, Faculty of Medicine, University of Malaya (UM), Kuala Lumpur, Malaysia; ^3^Medical Device and Technology Centre (MEDiTEC), Universiti Teknologi Malaysia, Malaysia

## Abstract

Minimal change disease (MCD) is the most common cause of idiopathic nephrotic syndrome in children, and it is well known for its multifactorial causes which are the manifestation of the disease. Proteinuria is an early consequence of podocyte injury and a typical sign of kidney disease. Steroid-sensitive patients react well with glucocorticoids, but there is a high chance of multiple relapses. CD80, also known as B7-1, is generally expressed on antigen-presenting cells (APCs) in steroid-sensitive MCD patients. Various glomerular disease models associated with proteinuria demonstrated that the detection of CD80 with the increase of urinary CD80 was strongly associated closely with frequent-relapse MCD patients. The role of CD80 in MCD became controversial because one contradicts finding. This review covers the treatment alternatives for MCD with the insight of CD80 as a potential therapeutic target. The promising effectiveness of CD20 (rituximab) antibody and CD80 inhibitor (abatacept) encourages further investigation of CD80 as a therapeutic target in frequent-relapse MCD patients. Therapeutic-based antibody towards CD80 (galiximab) had never been investigated in MCD or any kidney-related disease; hence, the role of CD80 is still undetermined. A new therapeutic approach towards MCD is essential to provide broader effective treatment options besides the general immunosuppressive agents with gruesome adverse effects.

## 1. Minimal Change Disease Background

Minimal change disease (MCD) is the most common cause of idiopathic nephrotic syndrome in children [1]. MCD is slightly more common in Asia and has a male predominance than woman (approximately 2 : 1) [2]. The majority of the nephrotic syndrome patients show MCD on histopathology diagnosis (80%) [3]. Observations by Shalhoub back in 1974 resulted in a strong relationship between the traditional view of MCD and circulating mediators produced by abnormal T cells [4]. One of the observations includes MCD is associating with Hodgkin disease and responding towards immunosuppressive drugs that suppress cell-mediated immunity. However, the exact pathogenesis of MCD is still unknown today [4, 5]. The theory of circulating mediators was supported by studies showing that supernatants of T cell hybridoma lines from MCD patients were able to trigger foot process effacement (podocyte injury) and proteinuria condition in a rat model [6]. There was vast evidence suggesting different aspects of T cell regulation and function for causing podocyte injury in MCD [5]. There were hypotheses for the pathogenesis of MCD with a central focus on podocytes that are shifted from T cells [7]. MCD and focal segmental glomerulosclerosis (FSGS) are the two most common causes of nephrotic syndrome in the pediatric population [8]. There was a high rate of disease relapse during adulthood, despite the availability of potent immunosuppressive agents [9]. It is crucial to develop novel MCD-based treatment because for the past few decades the main treatment options remain, and it is not beneficial to a certain group of patients.

Proteinuria is an early consequence of podocyte injury and is a typical sign of kidney disease [10]. Proteinuria is defined as the clinical presentation of MCD, a range of proteinuria in children > 40 mg/m^2/^h or >2 g/day, accompanied by hypoalbuminemia (serum albumin < 2.5 g) and hypercholesterolemia [11]. In adults, nephrotic range proteinuria is defined as proteinuria of more than 3.5 mg/day. Infections could trigger subsequent relapses of MCD [12]. Complete remission (CR) is defined as proteinuria < 166 mg/1.73m^2^/day or <4 mg/m^2^/h or urine protein/creatinine ratio < 0.2 mg/g for more than 3 days and serum albumin > 3.5. Partial remission (PR) is defined as proteinuria > 166 mg/1.73m^2^/day (<4 mg/m^2^/h) but less than 2 g/day (<40 mg/m^2^/day) and serum albumin > 2.5 g or reduction in proteinuria > 50% from baseline [1]. Steroid-sensitive nephrotic syndrome (SSNS) is the normalization of proteinuria within 4 weeks of initiation of a standard dose of oral prednisolone [1]. The relapse of nephrotic syndrome is a condition after an initial response, reappearance of proteinuria > 40 mg/m^2^/h for 3 consecutive days. Infrequent relapse is the reappearance of proteinuria less than 3 times in a year, while frequent relapse is the reappearance of proteinuria around two or more times within 6 months or 4 relapses in the year following the initial therapy [1]. Steroid resistance (SR) is a condition that fails to respond to daily oral prednisolone to induce inadequate remission doses after 4 weeks of treatment in children while in adults it is classified as steroid resistance after 16 weeks of no response towards oral prednisolone at a dose of 1 mg/kg/day within 16 weeks [1]. Different responses of MCD patients towards glucocorticoid medication and successfully staying in remission decided the chance of recovery and back to full health.

Podocytes are highly specialized cells that cover the glomerulus, as the critical unit cells of the filtration barrier in the glomerulus; normal kidney function relies on healthy podocytes [13]. Podocytes maintain the physiological stresses at the same time they adapt to preserve function. The cytoskeletal actin system maintains podocyte foot process (large body cell with major cytoskeleton-linked cellular processes [13]), and abnormalities of the podocyte actin system lead to podocyte effacement [14]. The inability to regenerate or proliferate of podocytes leads to a decrease in cell number, but podocytes display a remarkable ability to recover from effacement and to reform the interdigitating foot process after an effective pharmacological intervention [15]. As long as the podocytes are not lost, they manifest a remarkable ability to recover the foot process quickly; this ability matches the description of MCD [15]. This phenomenon of podocyte recovery gives hope to the therapeutic field as the mouse model displays rapid but reversible foot process effacement and proteinuria. Nevertheless, podocyte foot process effacement was reported not correlated with the level of proteinuria in human glomerulopathies [16].

## 2. CD80/B7-1

CD80, also known as B7-1, is generally expressed on antigen-presenting cells (APCs) in steroid-sensitive MCD patients. Various glomerular disease models associated with proteinuria have reported the detection of CD80 [17]. The activation of CD80 on APCs and its binding to the CD28 receptor on T cells have a crucial role in T cell activation [7]. The binding of CD80 to cytotoxic T lymphocyte-associated- (CTLA-) 4 terminates the T cell response [18] while the CTLA-4 is expressed on the membrane of the Foxp3+ T regulatory cell (Treg) and Treg may further inhibit the immune response by the release of soluble CTLA-4 which also has a function to suppress CD80 expression on APCs. CD80 expression is inhibited by CTLA-4 and IL-10, which caused the remission of the proteinuria [18]. The impaired function of Tregs was observed in MCD patients [19]. Lipopolysaccharide (LPS) induced the elevation of CD80 expression on podocytes by binding to Toll-like receptor- (TLR-) 4 in association with the development of proteinuria and foot process effacement. A similar condition of CD80 expression was also found in cultured podocytes with disorganization of the actin cytoskeleton [20]. T cell cytokines such as IL-13 and polyinosinic:polycytidylic acid could also induce the expression of CD80 [21]. It was verified by immunofluorescence assay that podocytes expressed CD80 with a molecular weight of 53 kDa, which is the same as that of CD80 on the membrane, rather than the soluble CD80, which is 23 kDa [22].

Clinical studies reported that CD80 is expressed in podocytes in MCD and FSGS [22–24], but these findings did not match the result of other clinical studies. In fact, in the studies of Novelli et al., the authors reported that B7-1 was not detected by the immunostaining method, yet B7-1 was still detected in infiltrating inflammatory cells, which provides a strong argument of the negative results [20]. Despite a study providing a controversial conclusion of CD80 not being detected, many studies argue its usefulness. One of the most reliable observations is that B7-1-deficient mice did not develop proteinuria after LPS injections, while the mice with severe combined immunodeficiency (SCID) became proteinuric and had increased B7-1 immunostaining on podocytes [25]. By using immunofluorescence, CD80 was observed primarily expressed on the surface of podocytes [22], based on the observation, since FSGS caused severe damage to the podocyte. Thus, the expression of CD80 was declined, which leads to urine CD80 that is less in MCD patients. Low level or zero levels of CD80 detection could be the reason why the study indicated that CD80 is only correlated with MCD but not FSGS [26]. In other words, healthy podocytes determine the amount of CD80 being expressed; the loss of podocytes also indicates that no level of CD80 is being expressed. CTLA-4, a protein receptor that binds with CD80 and downregulates the response of CD80, has been suggested as a potential treatment for glomerular disease [23].

The current treatment of chronic kidney disease mainly targets optimizing renal and heart risk factors instead of being kidney oriented [27]. The current treatment for MCD largely relies on immunosuppressant drugs by correcting lymphocyte dysfunction, especially T cells. Glucocorticoid was reported to be useful towards MCD patients mainly because of the glucocorticoid receptors on kidney cells [28]. CR is achieved in 80%–90% of children with MCD after treatment with an immunosuppressant, but the steroid mechanism is still unknown [7]. The responsiveness towards corticosteroids is different between individuals; adults often showed delayed response by 8–16 weeks to corticosteroids and approximately 65%–80% of adults with MCD will relapse within the first 3–6 months after remission [29, 30]. Moreover, 25–30% adults with MCD have frequent relapse; there is a substantial number of patients that have poor long-term result towards corticosteroids and usually suffer from frequent relapses which eventually lead to renal impairment [31, 32].

## 3. Treatment Options in MCD

*Prednisolone* (Table 1) is undoubtedly the most common treatment for MCD for both children and adults during early onset, while frequent-relapse patients will be given another drug to expect a remission. Oral prednisolone is given in a dose between 60 mg/m^2^/day and 80 mg/day for 6 weeks as a treatment for children with first episode of MCD while the adult is treated with prednisolone at a dose of 1 mg/kg/wt./day daily or 2 mg/kg/day on alternate days [1]. Prednisolone is also frequently reported to be used in combination with other agents as treatment options (Table 1). It was reported that single daily dosing is as effective as multiple daily dosing in maintaining remission in nephrotic syndrome children [33]. As part of the limitation of prednisolone in Table 1, its mode of mechanism to achieve remission of nephrotic syndrome is still unknown [34]. Various speculations of hypothesis explain the mechanism of prednisolone against MCD patients; most of it went beyond the typical anti-inflammatory or immunosuppressive actions because glomerular inflammation is mostly absent in SSNS [34].

*Levamisole* (Table 1) has been used as a treatment in some studies for MCD children [35]. Levamisole has promising results to maintain remission in children [36], but there is no evidence to explain this medication's mode of action at the molecular level (Table 1) [37]. Speculation of the effectiveness of levamisole could be the direct actions on podocytes [38]. Recent studies reported that levamisole significantly improves the relapse-free rate in children with frequent relapses or steroid dependency [39–41]; this outcome shows the ability of levamisole in preventing the relapses.

For the past 30 years, *cyclophosphamide* (CYC) and *chlorambucil* (CHL) as alternative of CYC have been used to treat children with relapsing SSNS (Table 1) [42]. A randomized trial showed that prolonging the duration from 8 to 12 weeks did not make any changes in relapses rate [43]. A preferred approach for frequently relapsing or steroid-dependent MCD in adults and children is the use of cyclophosphamide [44]. Oral cyclophosphamide at 1–2 mg/kg per day is initiated after reattaining a remission with prednisone [44]. However, the cumulative dose of cyclophosphamide should be monitored in the course of the given 8–12 weeks of medication because it is associated with adverse effect in Table 1 [45]. It was reported in another study that intravenous cyclophosphamide and oral prednisolone were as effective as the combination of intravenous dexamethasone together with cyclophosphamide and prednisolone oral therapy regarding its ability to induce remission in patients with steroid-resistant nephrotic syndrome [46]. In adults, cyclophosphamide or calcineurin inhibitors have been used with up to 75% response rate [47]. The nonrelapse rate for the CYC group was 60% compared with a 24% nonrelapse rate in the steroid group [48].

Compared to cyclosporine, *Mycophenolate Mofetil* (MMF) is a less toxic adjuvant agent (Table 1) for remission maintenance in childhood-onset relapsing or steroid-dependent MCD compared to cyclosporine [49–51] with the tendency towards a higher risk of relapse in patients treated with MMF [49]. Mycophenolate is shown to be a promising alternative treatment for adult MCD patients in case series [50, 52–54]. MMF was reported to be effective in lupus nephritis but not as effective in primary glomerulonephritis [53]. However, this outcome might be controversial with the studies of Dimkovic et al., as the authors reported that MMF proved to be efficient in 70% of high-risk patients with primary glomerulonephritis [54]. Studies by Fujinaga et al. (2009) suggested a similar result with Baglio et al. (2006) that MMF may not necessarily be associated with improved long-term outcomes or no beneficial effect for children with steroid-/cyclophosphamide-/cyclosporine-dependent/resistant nephrotic syndrome (Table 1). While rituximab is increasingly used in treating idiopathic steroid-resistant nephrotic syndrome in children, MMF may be an effective and safe maintenance therapy as an additional immunosuppressant among children with persistent steroid-resistant nephrotic syndrome [55].

*Cyclosporine A* (CsA) (Table 1) has been effective in treating and preventing frequent relapse of MCD with CR rates up to 80% [44]. CsA is relatively safe to use in developing chronic cyclosporine nephrotoxicity with or without other immunosuppressive agents [56]. Steroid-resistant patients benefit from CsA while steroid-sensitive patients have an alternative besides prednisolone. The combination of CsA and prednisolone can reduce the initial dosage of prednisolone, hence shortening the time to remission and easily maintaining it. This combination protocol indicated the effectiveness and usefulness and could potentially be a future treatment strategy for frequent-relapse nephrotic syndrome [57]. The most crucial drawback of CsA (Table 1) could be the high relapse rate after withdrawal of the medication.

*Tacrolimus* (Table 1) with high remission rates of up to 91% achieved in 2 years has been reported. Moreover, remission rates of up to 100% were reported in a few case series [29, 58, 59]. Tacrolimus could be a promising second option for cyclosporine in steroid-resistant and steroid-dependent nephrotic syndrome due to its high remission rate [60]. Tacrolimus was suggested to be used as a combined therapy [61] with the first reported case showing that sirolimus successfully resulted in remission in minimal change nephropathy [62].

*Dexamethasone* could recover podocyte injury by regulating podocytes' structure because it acts as the critical cytoskeletal protein anchoring the focal adhesion and slit diaphragm proteins that are responsible for podocyte attachment to both the underlying glomerular basement membrane and adjacent podocytes [63]. Table 1 shows the potential mechanism of why dexamethasone could increase the stability of the actin filaments hence reducing podocyte effacement by partially blocking the activation of Toll-like receptor 3 that induces the expression of CD80 and phenotypic change via an NF-*κ*B-dependent mechanism [64]. The knockdown of CD80 protected against actin reorganization and reduced synaptopodin expression [64].

*Rituximab* (Table 1) is an antibody against CD20, which upon administration will severely deplete B cells [65]. A study reported that overexpression of sphingomyelin phosphodiesterase acid-like 3b (SMPDL-3b) or treatment with rituximab could prevent disruption of the actin cytoskeleton and podocyte apoptosis [66]. An effective case series in Table 1 affirmed the role of rituximab in proteinuria condition; however, studies by Guigonis et al. (2008) were focusing on FSGS patients instead of MCD patients. Adverse effects were observed in ten cases by Guigonis et al. (2008) and also reported in Bagga's review that rituximab has serious adverse effects, in which it was only used if other agents fail to show the result [67]. In another study, it was suggested that the antiproteinuric effect of rituximab might be independent of B cell depletion [66]. Moreover, it was reported that rituximab could be useful for predicting relapse in patients with nephrotic syndrome [68] and sustained CR of steroid- and cyclophosphamide-resistant MCD patients with a single course of rituximab therapy was reported as well [69]. Nevertheless, among many other medications compared with rituximab with regard to the long-term remission, only rituximab was reported to successfully have long-term remission of MCD [70]. T helper cell 17 in Table 1 was reported to associate with reduced inflammation.

Nonetheless, it was reported that the annual relapse rate had been reduced by rituximab [71]. Additionally, rituximab could change the course of severe relapsing steroid-dependent nephrotic syndrome; evidence showed in a case studied [72] in which the authors speculated that rituximab could either directly affect or change the responsiveness to cyclosporine, but the attempt to reduce or withdraw cyclosporine should be monitored. With such shreds of evidence, anti-CD20 antibodies may be a promising therapy [73]. Controversial reports regarding the effectiveness against FSGS and MCD shall be investigated further with all researchers' efforts to report the actual value of rituximab and firm conclusion [74].

It was surprising that due to the rituximab resistance of patients of nephrotic syndrome, ofatumumab (Table 1) was given as treatment of multidrug resistance of chronic lymphocytic leukemia in a case study of a 19-year-old girl with a 6-year history of steroid-resistant nephrotic syndrome [75]. The therapy of ofatumumab had given a shocking response in the patient's renal status, in which proteinuria improved after only the first dose of ofatumumab [76]. Ofatumumab could be an effective treatment for steroid-resistant nephrotic syndrome, but more confirmation studies should be conducted for this observation. Ofatumumab was reported to enhance its efficacy in combination with chemotherapy and biological agents [75]. The response of steroid-resistant nephrotic syndrome towards rituximab and ofatumumab suggests a robust logical approach towards CD80 investigation and the potential effectiveness of anti-CD80 specifically against minimal change nephrotic syndrome.

*Abatacept* (cytotoxic T lymphocyte-associated antigen 4-immunoglobulin fusion protein (CTLA-4-Ig)) is a costimulatory inhibitor that targets B7-1 (CD80) [23]. There was evidence that abatacept (CTLA-4-immunoglobulin fusion protein) may both directly and indirectly support the fact that MCD patients could be impaired with Tregs by inhibiting the pathogenesis of rheumatoid arthritis at several levels via selective modulation of CD80/CD86 costimulatory molecules of MCD [7, 77]. A case report presented that abatacept was believed to have a fundamental role in maintaining disease remission and supported the value of CD80 in relapsing MCD patients [78]. In the case report by Isom et al., the longest successful usage of abatacept to treat a relapsing steroid-dependent MCD patient was reported. With that, the MCD patient had a successful reduced usage of both tacrolimus and prednisone.

*Mizoribine* (Table 2) was reported to significantly reduce the relapse rate and prolong the remission period of nephrotic syndrome [79]. Mizoribine is more commonly used than azathioprine as an immunosuppressive drug [80]. *Azathioprine* (Table 2) was reported as a cheaper alternative second to cyclophosphamide, and the efficacy might be restricted [81]. The studies on azathioprine were limited and did not prove with convincing results, but it should be reserved for future investigation.

*Galiximab* is an antibody against CD80 [82], but it was never tested with renal disease patients. Nonetheless, it could also be an option to further investigate its mechanism towards MCD patients. Galiximab is an anti-CD80 monoclonal antibody, besides abatacept, a CD80 targeting protein; nonetheless, abatacept is not the antibody of CD80 [83]. It was reported that galiximab had been used as a treatment for hematologic malignancy, such as relapsed non-Hodgkin's lymphoma [82, 84]. However, there is not much information regarding the effectiveness of galiximab, and certainly, it has never been used for any renal disease. Therefore, the research interest for galiximab remains for targeting podocyte and proteinuria.

*Pefloxacin* (Table 2) had antiproteinuric effect in a small group of patients which was reported, but later on it is not widely used or reported [85–87]. *Mechlorethamine* had been reported in the medication histories of some patients in a study against rituximab [86] as well as the effect of reducing relapse frequency in patients [88]. Limited information is available about the purpose of this medication in MCD; usually, it was used as an alternative of alkylating agents besides CHL and CYC.

*Basiliximab* (Table 2), an anti-interleukin-2 (IL-2), was reported to show effectiveness in some minimal change nephrotic syndrome patients [89]. The surprisingly significant effect of basiliximab shown in Table 2 provided good insight of the antibody's role in MCD. Although basiliximab's effectiveness was reported in a case study, it was insufficient to provide scientific logic towards the result because the patients also suffer from several disease- and treatment-associated complications such as indirect inguinal hernias, cataracts, and bacterial infections. Therefore, the mechanisms of basiliximab might vary, and further investigation should be performed.

**Figure 1:**
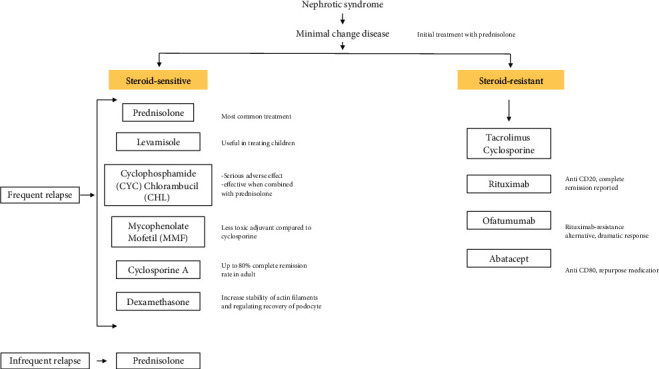


Figure 1 summarizes the commonly used medication in MCD. There were other potential treatment approaches reported such as the use of sirolimus, everolimus, adalimumab, fresolimumab, sparsentan, galactose, and thiazolidinediones summarized in Table 2. However, these approaches extremely lack strong evidence and are weakly associated with MCD. Most of the studies were not MCD focused. Nonetheless, their role in proteinuria condition should not be overlooked in this multifactorial disease. Multiple sclerosis-approved medication FTY720 which is an immunosuppressive agent for CD80 had been suggested as potential treatment of podocyte injury [83]; however, no further studies had been done in MCD patients.

## 4. Discussion

The recent finding suggested that the therapeutic targets for glucocorticosteroids, cyclosporine, and rituximab could be the molecules expressed by podocytes in MCD [15]. Novel therapeutic agents directed against these molecules may assist in the stabilization and reconstruction of podocytes in MCD. For instance, the suppression of CD80 expression on podocytes could be the therapeutic interest in MCD, FSGS, and glomerular disease [7]. Moreover, the actin cytoskeleton could be a therapeutic target as disorganization in the actin cytoskeleton architecture caused podocyte effacement (injury of podocyte foot process), which might represent the underlying molecular pattern for the morphogenetic transformation, and there was a study demonstrating that stabilizing the actin cytoskeleton is of therapeutic value [90].

A combination of treatments shown in Figure 1 shall be considered in this multifactorial disease (MCD) instead of monotreatment. The therapeutic target of MCD could be more than one at the same time. Therefore, it is challenging to pinpoint an absolute effective treatment against MCD. A multilayer of treatments shall be considered after screening prominent targeted markers shown in Tables 1 and 2. A combination treatment of antibodies and glucocorticoids should be investigated for its efficiency for relapse patients since a monotreatment approach of either glucocorticoids or inhibitor medication such as *abatacept* did not show a promising result. Combination treatment with *prednisolone* such as *rituximab* with prednisolone [91], *mycophenolate* with a low dose of prednisolone [92], and *cyclosporine* with prednisolone [93] had been studied but not CD80. Thus, a combination of CD80 antibodies with a low dose of prednisolone could be an excellent potential approach. The combination of prednisolone and cyclosporine treatment did show changes in overall response and should be an essential option of treatment [93]. The combination of mycophenolate with a low dose of prednisolone is also helping with better tolerability and fewer adverse effects [92]. Perhaps, the approach of a combination of medication could replace sole use of prednisolone.

The increase of urinary CD80 in SSNS relapse patients [94] shows the positive result with glucocorticoids at the beginning. However, the relapse happened with higher urinary CD80 level detected, giving reasonable speculation that CD80 should be targeted individually instead of giving generic immunosuppressive drugs because if the immunosuppressive drug is valid the relapse shall not happen. Hence, a specific antibody targeting MCD-caused CD80 expression should be developed instead of using a repurpose drug of rheumatoid arthritis (*abatacept*). Abatacept (B7-1 blocker) should not be rushed to be introduced as treatment until ongoing research about CD80 provides more information [20]. The treatment of the B7-1 blocker as a potential treatment remains controversial because it is not consistent with other studies [17].

Despite the evidence of some studies indicating that CD80 may not be valuable as a therapeutic target, studies by Reiser et al. and Khullar et al. showed that the induction of CD80 in podocytes might drive proteinuria and podocyte dysfunction [24, 95]. On the other hand, the researcher in Table 3 also found remarkable improvement from case studies of proteinuria in a child with recurrent MCD and a young adult gentleman with relapsing condition; both individuals were treated with abatacept [78, 96]. Evidence from Isom et al.'s studies is strongly encouraging that CD80 should remain as an attractive diagnostic and prognostic biomarker. Table 3 puts together a series of recent studies of CD80 in MCD from animal and human studies.  Table 4 summarizes the human studies of CD80; there were limited studies against the value of CD80. Nonetheless, due to the nature of the MCD being multifactorial, there are at least 76 different causes and associations which have been reported with regard to minimal change disease [97, 98]; keeping an open mind towards frequent-relapsing MCD patients that show increased level of CD80 as a therapeutic target will be beneficial than shutting the door of the possibility of its role in the pathogenesis of MCD. Many studies prove CD80 values as positive in MCD until Novelli et al. (2016) and Minamikawa et al. (2018) reported the negative finding of CD80 in MCD and FSGS patients which had created a polarization effect on the researcher. Nevertheless, let us be reminded again that MCD is a multifactorial disease; it could both be right (positive outcome and negative outcome); the focus should remain on the condition and environment that leads to the negative finding of it to understand the pathway better instead of weighting yes or no of the role of CD80. A negative finding of CD80 in MCD and FSGS patients only sparks the curiosity of the researcher to investigate more in-depth of its relevant pathogenesis backtracking to the origin of the disease, whether it is primary-caused MCD or secondary-caused MCD.

Studies that debate over CD80 as a nonreliable biomarker are understandable. In contrast, studies that show CD80 is associated with MCD are well evidenced, because there are far too many reasons and potential association to cause podocyte effacement, which manifests as minimal change disease. The reason could range from toxicity, immunology, charge distortion, signaling pathway activation, and genetic modification. Each of these categories manifests different mechanism and different pathogenesis related to MCD most of the time known as idiopathic MCD. Therefore, despite the ambiguous speculation of the role of CD80 in MCD, the importance of the role of CD80 in MCD indeed has been reported [22, 26, 64, 99–102]; hence, whether CD80 should be or should not be a therapeutic target indeed remains a colossal interest for the researcher to investigate further [103]. There might be a close link between CD80 and angiopoietin-like 4 (Angptl4) which is also a key player in the induction of proteinuria, but the relationship between Angptl4 and CD80 is not yet determined [104] because in a mouse model of MCD that was injured with LPS both the expressions of Angptl4 in adipose tissue [105] and CD80 on podocytes [24] increased.

## 5. Conclusion

Various fields have been explored, but the current clinical practice towards MCD patients still relies heavily on immunosuppressive medication. Podocyte indeed sparks a vast research interest, but again, it does not change the clinical practice towards MCD patients. The challenges of MCD always lie on unknown therapeutic target. The multifactorial nature of the disease should be treated with the screening of specific biomarkers before any treatments. Based on the positive outcome of the various medications in this review paper, we support the idea that elevated specific biomarkers could be targeted more effectively rather than general immunosuppressive medication. A personalized treatment starting from the screening of specific biomarkers shall be practiced from new emerging evidence in the studies of MCD. Future research shall focus on various in-depth biomarker patterns; thus, customization of MCD treatment plans could work. The positive study outcomes of CD20 (rituximab) medication in Table 1, also the inhibitor of CD80 (abatacept), trigger the interest and strong likelihood of investigating specific antibodies of CD80 for therapeutic effects. CD80 therapeutic (antibody) aspect was never clinically or thoroughly investigated; most of the research about CD80 was still debating the involvement of CD80 in MCD. The role of CD80 could be subtle or specific in certain conditions such as frequent relapse or the injury mechanism; again, MCD is a multifactorial disease. Hence, an effective target-specific antibody for CD80 that could reduce the effacement of podocyte's foot process and cease the proteinuria condition in a frequent-relapse MCD patient will ultimately prove the value of CD80. Yet, such studies are rare.

## Figures and Tables

**Table 1:** Commonly used medication in minimal change disease.

Treatments options	Description/usefulness	Drawback/limitation	Target molecule/cell
Prednisolone (glucocorticoids)	(i) Standardized treatment for MCD in children and adults [1, 11] (ii) Responsiveness of infant towards prednisolone as diagnosis of MCD without biopsy	(i) Dreadful adverse effect [34] (ii) Often used in combination with other medications (iii) Exact mode of action is unknown [2] (iv) Not useful for steroid-resistant MCD patients (v) Not designed for MCD patients	(i) Glucocorticoid receptor, T helper subtype 2 (Th2) cytokines such as interleukin-4 and interleukin-13 [34]

Levamisole (synthetic imidazole)	(i) Useful in children but lack molecular explanation [37, 106] (ii) Cheap, least toxic, and could prevent relapse [39]	(i) Availability issue [106] (ii) Extremely high relapse followed by discontinued treatment [106]	(i) Modulates type 1 and type 2 immune response by enhancing interleukin-18 [107] (ii) Glucocorticoid receptor's signaling as critical target of levamisole's action [37]

Cyclophosphamide (alkylating agent)	(i) The most effective and great experience in treatment for frequent relapse children and adults [1, 44] (ii) Higher rate of cumulative sustained remission compared with cyclosporine [108]	(i) Various severe side effects reported including bone marrow suppression, bladder toxicity, gonadal toxicity, malignancy, hemorrhagic cystitis, gastrointestinal disturbances, alopecia, and infection [1, 109]	(i) Unknown in MCD [110] (ii) Effective at reducing antibody production by B cell; T cells could be affected by the expression of aldehyde dehydrogenase [109]

Chlorambucil (alkylating agent)	(i) Second option after cyclophosphamide	(i) Greater toxicity without any improvement [1]	(i) Unidentified

Mycophenolate Mofetil (immunosuppressive agent)	(i) Less side effects than cyclosporine [49–51] (ii) Important effects on treatment course together with rituximab [111]	(i) More effective in lupus nephritis instead of MCD [53] (ii) No beneficial result for steroid-, cyclophosphamide-, and cyclosporine-resistant patients [112]	(i) Inosine monophosphate dehydrogenase (IMPDH) [51]

Cyclosporine A (calcineurin inhibitor)	(i) Effective in treating and preventing relapse in steroid-unresponsive children [113] (ii) Effective strategy to combine with prednisolone [57] (iii) The first option as immunosuppressant for treating children with refractory nephrotic syndrome [83]	(i) Optimal dose is unknown [2], but medium dose is safe for developing nephrotoxicity [56] (ii) Long-term outcome is unknown and may not beneficial to steroid-dependent nephrotic syndrome patients [114] (iii) High relapse rate right after medication withdrawal (iv) Hypertension as the most common adverse effect followed by increase of creatinine levels, hyperkalemia, gingival hyperplasia, and hypertrichosis [110, 115]	(i) Nuclear factor of activated T cell (NFAT) [116] (ii) Phosphorylates synaptopodin in podocyte (stabilization by binding to 14-3-3 site) [83]

Tacrolimus (calcineurin inhibitor)	(i) Promising second option of steroid and cyclosporine resistance with high remission rate [60] (ii) Combined therapy with sirolimus was effective [62] (iii) Tacrolimus is 10 to 100 times more potent than cyclosporine in its immunosuppressive effects [117] (iv) Lower relapse rate and cosmetic side effects compared to cyclosporine [115, 118]	(i) Worsen and new onset of hypertension reported [59] (ii) New onset of diabetes mellitus, infection was reported [59] (iii) Increased risk of diabetes mellitus [115]	(i) Inhibit T lymphocyte by binding with FKBP-12, calcium, calmodulin, and calcineurin [117]

Dexamethasone (glucocorticoid)	(i) Stabilize actin filament (reduce podocyte effacement) [119] (ii) Partially inhibit CD80 [64] (iii) Protect podocyte from apoptosis [120]	(i) Hypertension and hypokalemia were reported with intravenous dexamethasone administration [46] (ii) Worsened proteinuria condition was reported [121]	(i) RhoA activities [122] (ii) Apoptosis mechanism protein such as tumor suppressor protein p53, bcl-2 family protein, caspase-3, and apoptosis-inducing factor (AIF) [120] (iii) Upregulated nephrin and tubulin-*α*, downregulated vascular endothelial growth factor [28] (iv) Interleukin-6 and interleukin-8 suppressed by dexamethasone [28]

Rituximab (antibody)	(i) Immediate response for 11-year nephrotic syndrome patient to achieve remission (free from proteinuria) [123] (ii) Prevention of actin cytoskeleton disruption [66] (iii) Achieved remission from proteinuria was reported in many case series [111, 124] (iv) Safe and effective to induce and remain remission; serious side effects were uncommon [74, 125]	(i) Common adverse effects such as fever, chills, nausea; urticaria, orthostatic hypotension, and bronchospasm are rare; most of the adverse effects were during the first infusion [126] (ii) Not recommended in the guideline of therapy due to serious side effects [67]; 45% of cases reported different types of mild or transient adverse effects in a case series [124]	(i) Type 1 chimeric monoclonal antibody CD20 [66, 75] (ii) Relapse always associated with CD19 in FSGS patients (contradicts with other patients' CD19 levels and lack of established evidence) [124] (iii) T helper cell 17 (Th17) and interleukin-17 [127, 128]

Ofatumumab (antibody)	(i) Effective alternative of rituximab [75], fully humanized (ii) Dramatic positive response reported [76]	(i) Various toxicities were reported [75]	(i) Different epitope of CD20 than rituximab [75]

Abatacept (fusion protein)	(i) Effective outcome of rheumatoid arthritis repurpose medication on MCD patients was reported [7, 23, 78]	(i) Increased risk of bacterial infection [129]	(i) CD80 (B7-1) [23] (ii) Restore *Β*1 integrin activation [23, 83, 103]

**Table 2:** Less frequently used medication in minimal change disease.

Treatments	Description/usefulness	Drawback/limitation	Target molecule/cell
Mizoribine (imidazole)	(i) Reduced relapse rate and prolonged remission were reported [130] (ii) Provides protective effects against CsA nephrotoxicity [131] (iii) Replacement immunosuppressive agent of azathioprine due to lower toxicity for childhood nephrotic syndrome and steroid-resistant nephrotic syndrome [80]	(i) Not widely used due to low efficacy [80] (ii) Not recommended for use in children [132]	(i) Inhibition of DNA synthesis, specifically to lymphocyte proliferation [80]

Azathioprine (imidazole)	(i) Complete remission was reported but only in 1 patient out of 20 [30]	(i) Not effective to prevent relapse in nephrotic syndrome children [133] (ii) Ineffective result had been noted [134] (iii) Not well documented [135] (iv) Not recommended for use in children [132]	(i) Inhibition of DNA synthesis, specifically to lymphocyte [136]

Pefloxacin	(i) Disappearance of proteinuria reported in frequent relapse nephrotic syndrome patient [87]	(i) Extremely limited evidence and studies, result only based on 1 patient [86] (ii) Toxicity towards joints, ankle, knee, and neck was reported [87]	(i) Unclear [85], lack of studies in nephrotic syndrome

Mechlorethamine (alkylating agent)	(i) Reduce the frequency of relapse in patients [88]	(i) Extremely limited evidence and studies, result only based on less than 5 patients [86]	(i) Unclear, lack of studies in nephrotic syndrome

Sirolimus (mammalian target of rapamycin (mTOR) inhibitor)	(i) Suggested as potential approach [2] (ii) Been used together with cyclosporine in 2 patients but the result is unclear [100] (iii) More commonly used in kidney transplant patients	(i) Anemia as commonly noted side effect [137], the most common side effect is hyperlipidemia [62] (ii) Studies showed sirolimus itself might have caused proteinuria [138] (iii) Most studies were FSGS related instead of MCD	(i) Inhibit proliferation of T lymphocytes, fibroblast, endothelial, mesangial, and smooth muscle cells [138]

Galiximab (antibody)	(i) Potential approach	(i) Never tested or used in kidney-related disease	(i) CD80 [82]

Basiliximab (antibody)	(i) Effective single dose to achieve complete remission and remission was reported [70, 139]	(i) Addition of basiliximab to treatment did not improve clinical outcome [140]	(i) Interleukin-2 [89, 139] (ii) CD25 [70]

Adalimumab (antibody)	(i) Subgroup that is TNF associated could be partially benefited [141]	(i) Studies conducted but no available result [15] (ii) Not suggested further in resistant FSGS [141] (iii) Studies in FSGS patients, lack of MCD focused	(i) Tumor necrosis factor-*α* (TNF-*α*)

Galactose	(i) Proteinuria reduction and kidney function preservation were reported [141] (ii) Remission of nephrotic syndrome in multiple immunosuppressant-resistant patient was reported [142]	(i) Studies conducted but no available result [15] (ii) Studies in FSGS patients, lack of MCD focused	(i) High affinity with FSGS permeability factor [142]

Thiazolidinediones (synthetic peroxisome)	(i) Evidences of reduced proteinuria, microalbuminuria, podocyte injury, vascular injury, inflammation, and fibrosis were reported [110] (ii) Podocyte protective feature was noted, could possibly be the potential therapy due to the similar response of glucocorticoids [110]	(i) Most studies focus on diabetic nephropathy and nondiabetic glomerulosclerosis, lack of studies in MCD patients	(i) Activation of the glucocorticoid receptor [110] (ii) Decrease transforming growth factor production [143]

Everolimus (mTOR inhibitor)	(i) Cytoskeleton stabilizing effect reported for the first time in 2013 [144] (ii) Lack of studies, especially in MCD	(i) Potentially worsen kidney's health and proteinuria [145] (ii) Lesser data than sirolimus [146] (iii) Mostly renal transplant focused instead of MCD	(i) Inhibition of T cell proliferation (ii) RhoA signaling [144]

Fresolimumab	(i) Partially achieved remission and reduce proteinuria in FSGS [147]	(i) FSGS patient studies, not MCD	(i) Transforming growth factor-beta [147]

Sparsentan	(i) Reduced proteinuria was reported [148]	(i) FSGS patient studies, not MCD	(i) Endothelin type A and angiotensin II type 1 receptor [147]

**Table 3:** Studies of CD80 in minimal change disease that are support or against in both animals and humans.

Animal studies	Stance on CD80	Human studies	Stance on CD80
Reiser et al., 2004 [24]	Support	Garin et al., 2009 [22]	Support
Shi et al., 2011 [149]	Support	Garin et al., 2010 [100]	Support
Ishimoto et al., 2013 [102]	Support	Ishimoto et al., 2013 [101]	Support
Novelli et al., 2016 [20]	Against	Yu et al., 2013 [23]	Support
Rivard et al., 2018 [150]	Support	Cara-Fuentes et al., 2014 [26]	Support
Khullar et al., 2018 [95]	Support	Novelli et al., 2016 [20]	Against
		Mishra et al., 2017 [151]	Support
		Liao et al., 2017 [94]	Support
		Minamikawa et al., 2018 [152]	Against
		Zhao et al., 2018 [153]	Support
		Ling et al., 2018 [8]	Support
		Ahmed et al., 2018 [154]	Support
		Bhatia et al., 2018 [155]	Support
		Isom et al., 2019 [78]	Support
		Cara-Fuentes et al., 2020 [156]	Support
		Chen et al., 2020 [157]	Support
		Gonzalez et al., 2020 [158]	Support

**Table 4:** CD80 in human studies' summary.

Studies	Type of studies	Subject number	Main finding	Conclusion
Garin et al., 2009 [22]	Clinical studies	MCD patients: *n* = 19 Control subjects: *n* = 9 FSGS patients: *n* = 4	Urinary CD80 was significantly higher in relapse MCD patients than MCD in remission.	Urinary CD80 is elevated in idiopathic MCD and relevant to diagnosis and prognosis.
Garin et al., 2010 [100]	Clinical studies	MCD patients: *n* = 17 FSGS patients: *n* = 22	CD80 is not elevated in FSGS patients.	CD80 may be a useful marker, supported hypothesis that MCD and FSGS were two different diseases.
Ishimoto et al., 2013 [101]	Basic science studies	Human podocyte cell line	An impaired CTLA4 response, the rapid upregulation of CTLA4 in glomeruli could be responsible for the transient CD80 expression.	CD80 production in podocytes with transient proteinuria; CD80 was relevant in the pathogenesis of proteinuria in MCD.
Yu et al., 2013 [23]	Clinical and basic science studies	FSGS patients: *n* = 5	CD80 could be a useful biomarker in the treatment of some glomerulopathies.	Abatacept induced complete or partial remission of proteinuria in patients.
Cara-Fuentes et al., 2014 [26]	Clinical studies	MCD: In relapse: *n* = 20 In remission: *n* = 21 FSGS patients: *n* = 26	FSGS patients have significant higher level of suPAR than relapse-MCD patients which showed correlation with proteinuria.	Urinary CD80 is elevated in MCD patients compared with FSGS patients.
Novelli et al., 2016 [20]	Clinical and basic science studies	MCD patients: *n* = 15 FSGS patients: *n* = 16 Male Balb/c mice for basic science studies	The therapeutic effect of abatacept might not be because of podocyte CD80.	Podocyte CD80 was not observed in mice or patients of MCD and FSGS.
Mishra et al., 2017 [151]	Clinical studies	Nephrotic syndrome patients: *n* = 70 Healthy controls: *n* = 23	MCD patients had higher median in expressing potential biomarkers than FSGS patients but no significant difference.	Urinary creatine/CD80 could be useful biomarkers in steroid-sensitive nephrotic syndrome in relapse.
Liao et al., 2017 [94]	Clinical studies	Nephrotic syndrome patients: *n* = 128 Healthy controls: *n* = 25	Higher urinary CD80 in recurrent phase steroid-sensitive nephrotic syndrome than healthy control or remission patients.	Urinary CD80 was strongly associated with relapse nephrotic syndrome but cannot be used as frequency of relapse prediction.
Minamikawa et al., 2018 [152]	Clinical	MCD patients: *n* = 31 FSGS: *n* = 9 Healthy controls: *n* = 30	Urinary CD80 was present in all active chronic kidney disease. Urinary CD80 was correlated with the urinary protein levels.	Urinary CD80 was an unreliable biomarker to differentiate relapse MCD and FSGS.
Zhao et al., 2018 [153]	Clinical	MCD patients: *n* = 55	CTLA4 absent or in minimum amount could distinguish steroid-sensitive MCD patients from steroid-resistant MCD patients.	Glucocorticoid was useful to result complete remission only in MCD patients, with strong CD80 level and minimum level of CTLA4.
Ling et al., 2018 [8]	Clinical	Nephrotic syndrome patients: *n* = 64	Urinary CD80 level could affect the response towards the initial treatment of steroid. High urinary CD80 level reacted 100% at the initial steroid treatment.	Urinary CD80 could predict the progression and remission of MCD in children, while also able to identify high-risk patients at an early stage.
Ahmed et al., 2018 [154]	Clinical	MCD patients: *n* = 21 FSGS patients: *n* = 9 Other glomerulopathies: *n* = 6 Healthy controls: *n* = 40	Urinary CD80 in MCD was significantly higher than FSGS and other glomerulopathies.	Urinary CD80 was significantly higher in MCD child patients.
Bhatia et al., 2018 [155]	Clinical	Steroid-dependent nephrotic syndrome patients: *n* = 18	The first study to report the effect of rituximab on urinary CD80 excretion.	Reduced urinary CD80 was observed after rituximab therapy.
Isom et al., 2019 [78]	Clinical case study	MCD patients: *n* = 1	The longest successful abatacept treatment that has ever been reported; 6 years of follow up, abatacept tremendously changed the pattern of relapse.	Strongly encourage the investigation of urinary CD80 as therapeutic and potential treatment target.
Cara-Fuentes et al., 2020 [156]	Clinical and basic science studies	MCD patients: *n* = 9 FSGS patients: *n* = 11	Relapse MCD patients had less CTLA4+ in glomeruli which caused the imbalance ratio of CD80/CTLA4 locally. There was a link between CD80 and endothelial cell activation. A second hit to the glomerulus could result in more significant podocytes injury and proteinuria.	Both podocytes and endothelial cells could be the potential sources of CD80 in human and animal model.
Chen et al., 2020 [157]	Clinical	MCD patients in relapse: *n* = 10 MCD patients in remission: *n* = 9 Healthy controls: *n* = 9	No correlation between urinary CD80 and proteinuria in adult onset MCD. Urinary CD80 was not a reflection of proteinuria.	Imbalance level of Th1/TH2/TH17 and elevated CD80 could be the pathogenesis of developing adult onset MCD.
Gonzalez et al., 2020 [158]	Clinical	MCD patients: *n* = 53 FSGS patients: *n* = 43 Healthy controls: *n* = 34	Urinary CD80 could serve as predictive marker for the potential responsiveness towards specific immunosuppressive agents.	Urinary CD80 could discriminate MCD from other nephrotic syndrome diseases.

## Additional Points

*Review Criteria*. The review was based on the search of Google Scholar, PubMed, Web of Science, and Scopus with a combination of search terms including the following: “minimal change disease”, “treatment options”, “CD80”, “nephrotic syndrome”, “frequent-lapse”, and “proteinuria”. No date or language restriction was set during the search.
